# CHAP: A Versatile Tool for the Structural and Functional Annotation of Ion Channel Pores

**DOI:** 10.1016/j.jmb.2019.06.003

**Published:** 2019-08-09

**Authors:** Gianni Klesse, Shanlin Rao, Mark S.P. Sansom, Stephen J. Tucker

**Affiliations:** 1 Clarendon Laboratory, Department of Physics, University of Oxford, UK; 2 Department of Biochemistry, University of Oxford, UK; 3 OXION Initiative in Ion Channels and Disease, University of Oxford, UK

**Keywords:** ion channel, channel regulation, hydrophobic gating, functional annotation, de-wetting, CHAP, Channel Annotation Package, MD, molecular dynamics, AMISE, asymptotic mean integrated square error

## Abstract

The control of ion channel permeation requires the modulation of energetic barriers or “gates” within their pores. However, such barriers are often simply identified from the physical dimensions of the pore. Such approaches have worked well in the past, but there is now evidence that the unusual behavior of water within narrow hydrophobic pores can produce an energetic barrier to permeation without requiring steric occlusion of the pathway. Many different ion channels have now been shown to exploit “hydrophobic gating” to regulate ion flow, and it is clear that new tools are required for more accurate functional annotation of the increasing number of ion channel structures becoming available. We have previously shown how molecular dynamics simulations of water can be used as a proxy to predict hydrophobic gates, and we now present a new and highly versatile computational tool, the Channel Annotation Package (CHAP) that implements this methodology.

## Highlights

• Some ion channels exhibit hydrophobic gating via dewetting of the central pore.
• This cannot be predicted from their pore radius alone.
• The hydrophobicity of a pore contributes to this dewetting effect.
• We have developed a new tool (CHAP) that combines these measurements.
• CHAP now enables the rapid functional annotation of ion channel structures.

## Graphical abstract

**Figure:**
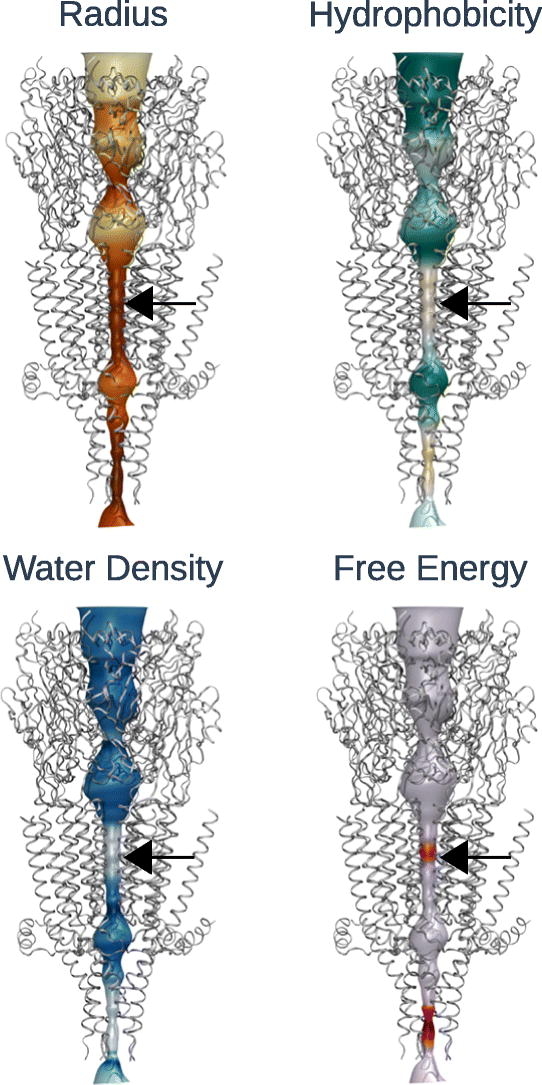

## Introduction

Ion channels form pores that facilitate the selective movement of ions across lipid bilayers. They therefore control a wide range of vital physiological functions and represent an important class of therapeutic targets. However, these pores are also highly dynamic structures that switch between functionally closed and open states in response to different physiological stimuli, a process referred to as gating [1].

**Fig. 1:**
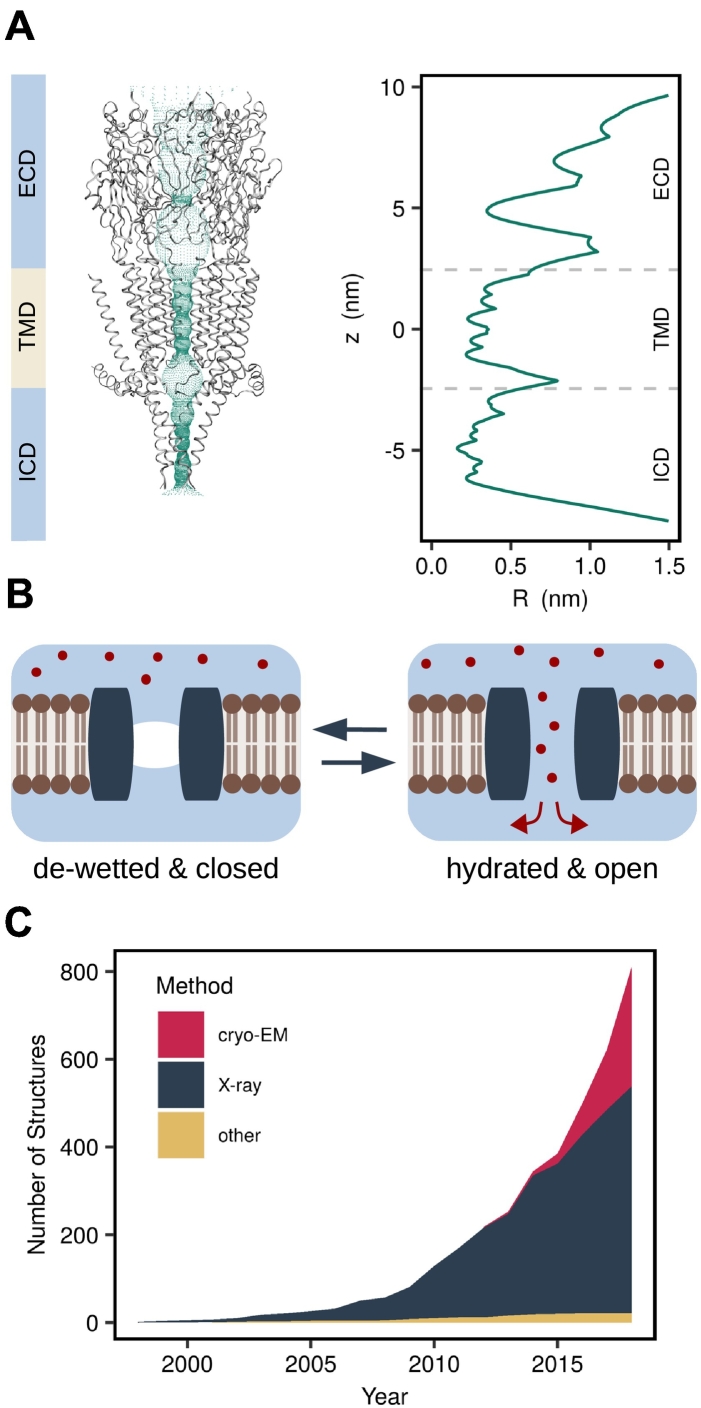
Hydrophobic gates pose a challenge to the functional annotation of ion channel structures. (A) HOLE profile of the entire PDB structure of a 5-HT_3_ receptor (PDB ID: 4PIR). The color bar indicates the extracellular (ECD), transmembrane (TMD), and intracellular (ICD) domains. (B) Hydrophobic gating: A non-conducting state is characterized by the de-wetting of a part of the permeation pathway lined by hydrophobic residues. Ions are prevented from crossing this region by the free energy barrier associated with their desolvation even if the pore is sufficiently large to accommodate them. (C) The number of ion channel structures deposited in the Protein Data Bank is growing exponentially (data retrieved on 5 December 2018).

Elucidating the molecular mechanisms that underlie these gating processes remains an important objective for ion channel structural biology and recent advances in techniques such as cryo-electron microscopy are now generating an increasing wealth of new ion channel structures, including many captured in different conformational states (Fig. 1). However, before this structural information can be compared to the detailed electrophysiological data that can often be obtained for many ion channels, the conductive status of any new structure needs to be known. Conventionally, this has been achieved by determining the physical (i.e., steric) dimensions of the channel pore. A variety of approaches and biomolecular visualization tools exist for determining the dimensions of pores, tunnels and cavities in proteins [2], but by far the most common software used for mapping transmembrane ion channel pores are programs such as CAVER [3], [4] and HOLE [5]. These calculate a radius profile along the conduction pathway and the structure is typically considered to be open and conductive if its narrowest constriction exceeds the radius of the hydrated ion species it conducts (Fig. 1A).

This size-based approach has served the ion channel community well for several decades, but an increasing body of evidence has demonstrated that this approach is unable to detect gates that are not based on steric occlusion of the permeation pathway. For example, “hydrophobic gates” can occur in channel pores to prevent ion flow and rely on the capillary evaporation of water that occurs when the lining of the pore is particularly hydrophobic [6]. In these situations, ion movement through these de-wetted sections of the pore is prevented by the free energy barrier associated with stripping the ions of their hydration shell (Fig. 1B) rather than by van der Waals clashes with tightly packed pore residues [7]. It is important to note that this definition of hydrophobic gating excludes cases where pore de-wetting immediately precedes the collapse and/or constriction of hydrophobic pore segments involved in steric closure due to a conformational change in the protein [8].

The phenomenon of hydrophobic gating was first observed in simulations of simple model pores [9], [10], [11] and has been confirmed experimentally in nanopores [12]. It has been found to occur within pores up to 1.2 nm in diameter and so includes the dimensions of most ion channel pores [13]. Therefore, an ion channel can be functionally closed even if the pore appears physically wide enough to accommodate the relevant hydrated ion.

The first possible example of a hydrophobic gate in an ion channel was in an early cryo-EM structure (PDB ID 1OED) of the nicotinic acetylcholine receptor, which suggested a functionally closed state without being sterically occluded [14]. The subsequently refined structure [15] of the same channel (PDB ID 2BG9) also suggests hydrophobic gating. Further examples of hydrophobic gates and barriers include the MscL mechanosensitive channel [16], the cation-selective bacterial channel GLIC [17], the BK and TWIK-1 potassium channels [18], [19], the BEST1 chloride channel [20], and the CorA magnesium channel [21]. Thus, hydrophobic gating clearly represents an important mechanism for regulating ion channel permeation and needs to be taken into consideration when assessing the functional status of any new ion channel structure.

In a previous study, we have shown how molecular dynamics (MD) simulations of water behavior within these nanoscale structures can be used to aid the functional annotation of different pentameric ligand-gated ion channels (the 5-HT_3_ and glycine receptors) and demonstrated the existence of hydrophobic gates within these particular structural conformations [22]. In particular, we established that pore hydration observed in simple equilibrium MD simulations can be used as a reliable proxy for ion permeability. This was validated by comparison to more computationally expensive and time-consuming approaches such as umbrella-sampling free energy calculations and computational electrophysiology simulations. Here we now present a new and highly versatile computational tool for use within the structural biology community that exploits the principle of pore hydration to rapidly predict the conductive status of new ion channel structures.

## Workflow for “Channotation” of Pore Structures

**Fig. 2:**
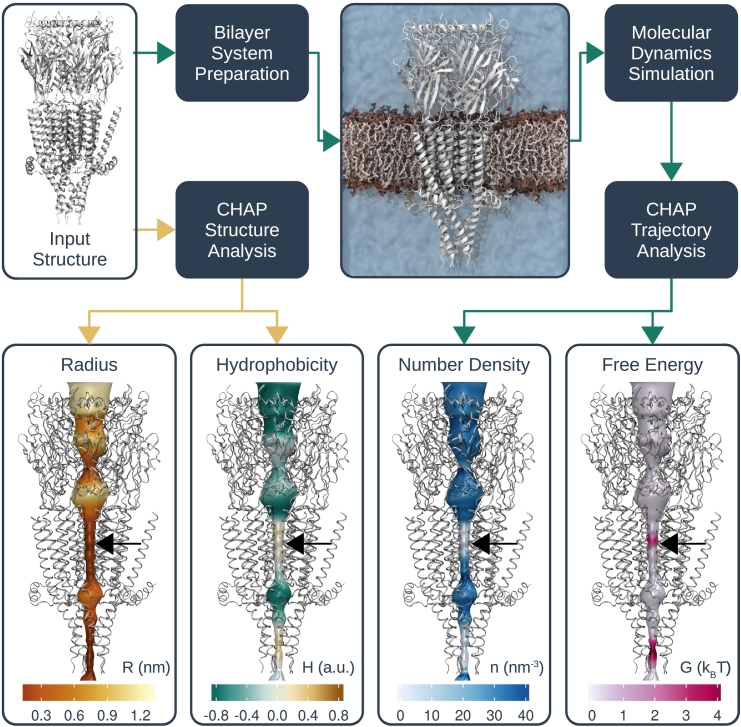
Overview of workflow for functional annotation of ion channels with CHAP. Given a simple protein structure as input, CHAP can calculate pore radius and hydrophobicity profiles. In addition, the protein can be simulated in its native lipid bilayer environment in order to assess the behavior of water inside the channel pore. From the resulting simulation trajectory data, CHAP can derive water density and free energy profiles that aid the identification of hydrophobic gates. Yellow arrows indicate steps of the pipeline that can be carried out on structural data alone, while cyan arrows indicate those steps that are based on subsequent MD simulation. The black arrows indicate the location of the 9′ ring of leucine residues in the middle of the hydrophobic gate in the transmembrane domain of the channel. There is a further free energy barrier within the intracellular domain, which coincides with a narrowing of the radius of the pore. In an open structure of the 5-HT_3_ receptor, ions are thought to permeate lateral portals within the intracellular domain, thus bypassing this constriction.

The workflow underlying this Channel Annotation Package (CHAP) is shown in Fig. 2, and the required software is freely available (www.channotation.org). As an initial analysis step, CHAP can be run directly on the PDB structure of any channel or nanopore to generate both radius and hydrophobicity profiles of the putative ion conduction pathway. These initial steps are easily performed on an average desktop computer and do not require MD simulation of the structure. However, if these profiles suggest that the channel may contain a hydrophobic gate or any other interesting features, then a more in-depth analysis based on equilibrium MD simulation of the structure can be performed.

The example illustrated in Fig. 2 is a crystal structure of a mammalian 5-HT_3_ receptor (PDB ID: 4PIR) in which the existence of a hydrophobic gate has been demonstrated by previous studies [22], [23], [24]. Initial structural analysis with CHAP (i.e., without simulation) yields plots of both radius and hydrophobicity for the entire channel pore. The simple radius plot shows the size of the transmembrane section of the pore to be roughly constant at ∼ 0.3 nm radius; however, the accompanying hydrophobicity plot also reveals this section to be somewhat hydrophobic, especially in the region of the 9^′^ ring of side chains.

To test for the presence of a hydrophobic gate within this region, the structure was inserted in a lipid bilayer system ready for equilibrium MD simulations using GROMACS. Briefly, the starting structure was embedded in a DOPC bilayer using a serial multiscale procedure [25], and the resulting protein-bilayer system was then simulated using the CHARMM36 all-atom force [26] field and the TIP3P water model [27]. The C*α* atoms of all protein residues were harmonically restrained at their positions in the experimentally determined structure (see the Supplemental Information for details of the simulation parameters). These restraints prevent any gating transitions or other large-scale conformational transitions, while still permitting, for example, side-chain reorientation. This ensures that any functional annotation produced by CHAP is as relevant as possible to the input structure itself, while simultaneously avoiding an unphysically rigid pore surface.

**Fig. 3:**
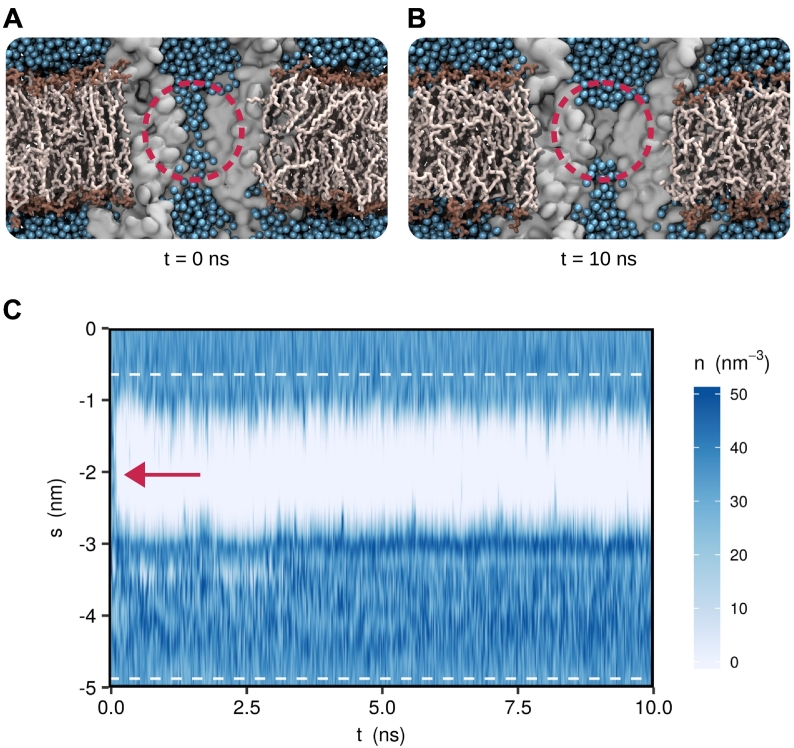
Hydrophobic gates can be identified through equilibrium MD simulation. (A) Cross section of the starting structure for MD simulation of a 5-HT_3_ receptor (PDB ID: 4PIR) embedded in a DOPC lipid bilayer. The protein is represented by a gray surface with two of five subunits omitted for visual clarity. Only the transmembrane (TM) domain is shown. Water oxygens are represented by blue spheres, and lipid molecules are shown in liquorice representation. Note that the entire channel pore is initially filled with water. (B) Snapshot from the MD trajectory clearly exhibiting a de-wetted region (highlighted with red circle) in the TM domain of the channel. Note that the channel pore is not physically occluded. (C) Water density profile in the receptor pore over time. An approximately 2-nm-long region of the TM domain indicated by a red arrow de-wets at the beginning of the simulation and remains devoid of water throughout.

As shown in Fig. 2, this allows calculation of the water density within the pore together with a free energy profile for hydration of the pore. The time-dependent behavior of water during this particular simulation is also displayed in Fig. 3. This shows that although the pore is fully hydrated in the initial configuration of the simulation system, water is expelled almost immediately (within ≪ 0.5 ns), leaving a 1 nm region of the conduction pathway fully dehydrated. Consistent with our previous studies [10], this rapid de-wetting was also observed within the first few nanoseconds in three independent MD simulations, each of which was run for a total of 100 ns. However, the rapid (<10 ns) nature of this dewetting indicates that much shorter simulation times are likely to be sufficient, and in this case, three 30 ns repeats would reveal a similar result. As discussed above, it is important to note that CHAP is designed to annotate specific experimentally determined structural states; that is, it is not intended to simulate major conformational rearrangements. Thus relatively short simulation times are likely to be sufficient. Using this approach and the profiles calculated by CHAP, it becomes immediately apparent that the transmembrane pore of this particular conformation of the 5-HT_3_ receptor contains a hydrophobic gate (Fig. 2). This would not have been revealed if only a radius profile such as that shown in Fig. 1A had been considered.

In addition to the examples described above, we have also applied CHAP to a recently solved structure of TRPV4, a member of the TRP family of cation channels where hydrophobic gating has also recently been suggested [28]. CHAP analysis of this structure reveals that although the lower gate may be able to pass hydrated Na^+^ and K^+^ ions, this region is also highly hydrophobic, thus presenting a major energetic barrier to hydration (Fig. S1). This supports previous reports of hydrophobic gating in TRP channels [29], [30] and suggests that further investigation of this effect in TRPV4 is warranted.

## Implementation of CHAP

Thus, although tools that calculate pore radius alone have proven exceptionally useful, it is clear that additional information is required if more accurate predictions are to be made. However, a simple extension of the popular HOLE program with added functionality was not practical for several reasons. In particular, HOLE was originally developed for the analysis of static protein structures and is unable to read common trajectory data formats or process time-dependent structures similar to those generated by MD simulation. We therefore decided to implement this new channel annotation package as a completely new and independent tool: CHAP is written in C ++ and is based on the trajectory analysis framework of the popular MD simulation software GROMACS [31].

**Fig. 4:**
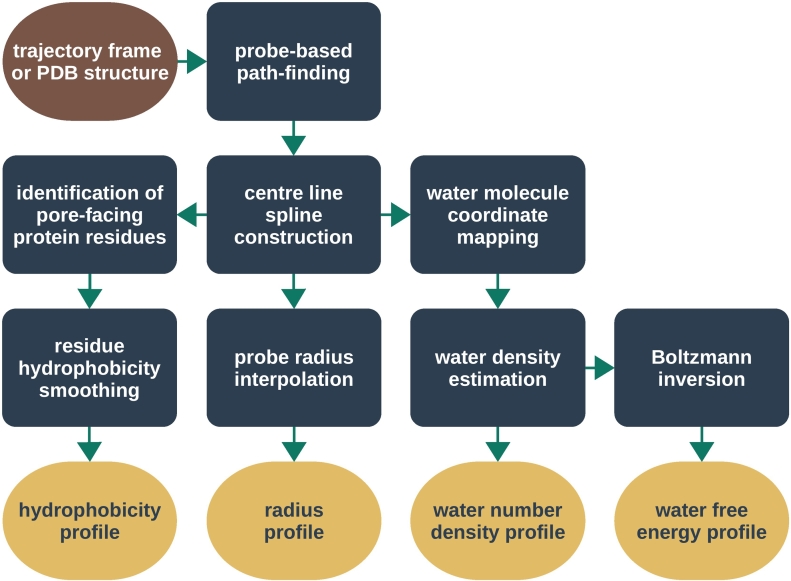
Workflow diagram of data processing in CHAP. For an individual PDB structure, this procedure is carried out once. For trajectory data, the above procedure is carried out individually for each frame, that is, for each structural configuration at a sampled time point. The resulting hydrophobicity, radius, density, and free energy profiles are averaged over all time steps, and confidence bands are estimated from the standard deviation over time.

**Fig. 5:**
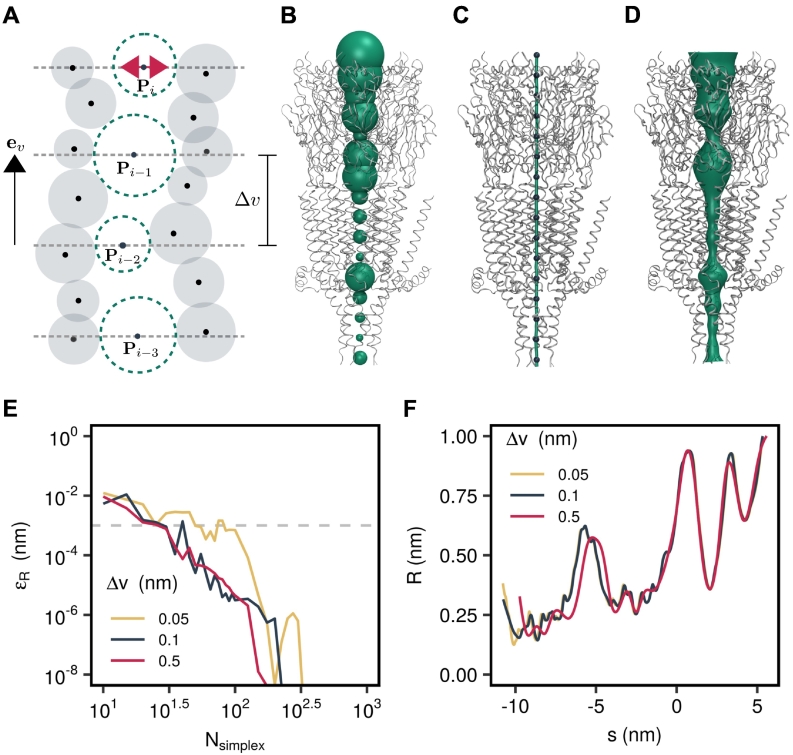
Pore geometry calculations in CHAP. (A) Schematic representation of probe-based pathway finding. The position of a spherical probe is optimized in subsequent parallel planes to maximize its radius without overlapping the van der Waals sphere of any ion channel atom. (B) Configuration space view of probe positions (green spheres) in the permeation pathway of a 5-HT_3_ receptor structure (PDB ID: 4PIR). For visual clarity, a large probe step of *v* = 1 nm was used here. (C) The probe center positions (blue spheres) are interpolated with cubic B-splines to yield a continuous spatial curve (green tube) representing the pore center line. (D) A surface representation of the channel pore is generated by extruding a circular cross section along the center line spline curve. (E) Convergence of the Nelder–Mead algorithm carried out to optimize the probe position in each parallel plane. The radius error falls below a threshold of 0.001 nm within 100 optimization steps irrespective of the value of the probe step. The reference radius profile is calculated with a very large number of 2000 Nelder–Mead iterations. (F) Influence of probe step on radius profile resolution. While a probe step of 0.5 nm only captures the general feature of the radius profile, decreasing the probe step from 0.1 to 0.05 nm leads only to minimal changes.

The algorithm used by CHAP to calculate these different channel profiles is outlined in Fig. 4. Starting from an input structure (either an experimentally derived PDB file or a single frame from an MD trajectory), the first step is to define the physical dimensions of the pore. A probe-based method is employed, similar to that used by HOLE, resulting in a sequence of discrete probe positions and associated pore radii. A continuous spatial curve is then determined from these probe positions by means of B-spline interpolation. A continuous radius profile is then calculated by interpolating the probe radii along this curve (Fig. 5).

**Fig. 6:**
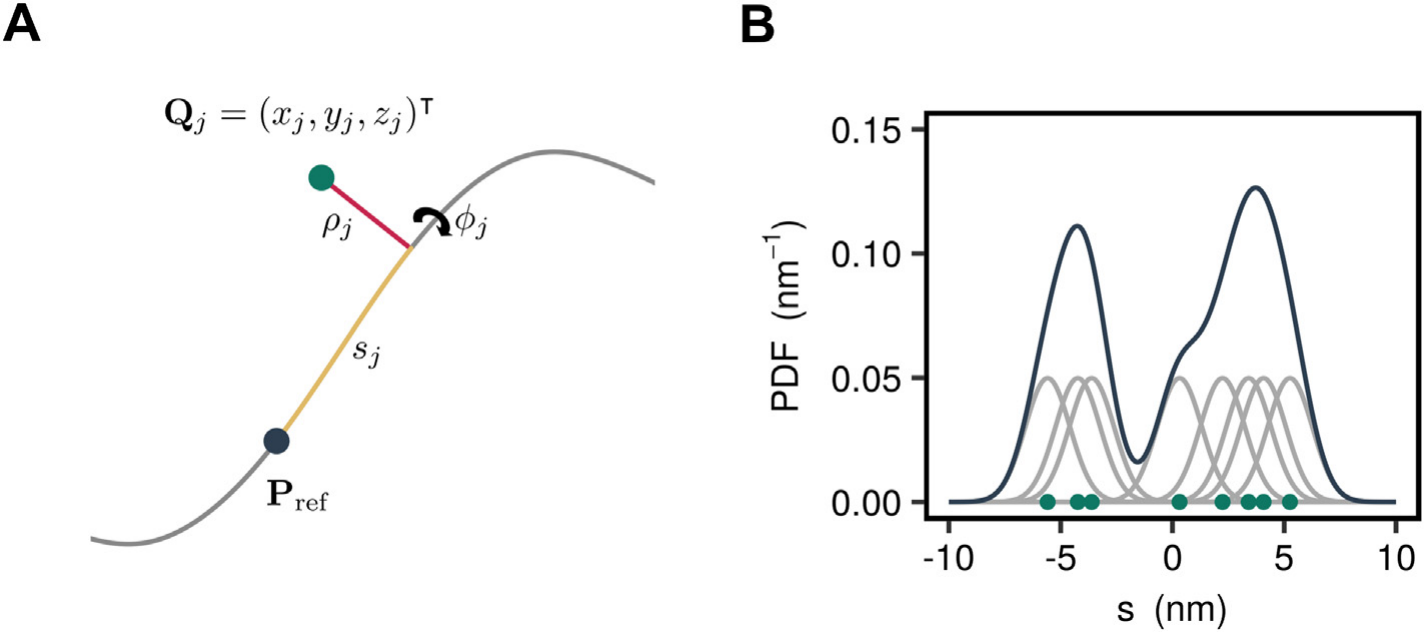
Determination of water density. (A) Cartesian coordinates of a water molecule (green) are converted into a curvilinear coordinate system by projecting its position onto the channel center line (gray). The new coordinates are then expressed in terms of the distance along the center line (yellow) between the projection base point and a reference point (dark blue), the distance from the water molecule to the center line (red), and an angle around the center line. (B) Conceptual representation of kernel density estimation: Each discrete water molecule position is associated with a Gaussian probability distribution and the sum of all Gaussians yields the probability density function of water along the channel center line.

This interpolating spline curve represents the center line of the pore and defines a coordinate system that forms the basis for all subsequent calculations. In this channel coordinate system, the position of a particle (e.g., a water molecule or a protein side chain) is represented by its distance to the closest point on the center line and the distance along the center line between this point and a reference point (Fig. 6A). By convention, the reference point is the projection of the center of geometry of the overall channel protein onto the center line.

Next, a hydrophobicity profile is determined for the residues that line the pore. By converting residue positions to channel coordinates, CHAP identifies all residues within a cut off distance from the pore surface and then selects only those whose side chains point inwards toward the pore. The resulting discrete values for hydrophobicity at the positions of the individual residues are then converted into a continuous hydrophobicity profile along the center line by means of kernel smoothing [32].

If the input structure contains water molecules, for example, from an MD simulation, then CHAP will also estimate the solvent density along the center line. To achieve this, water molecule positions are first transformed into channel coordinates, and then a one-dimensional probability density is calculated by kernel density estimation [33]. Together with the radius profile, this allows us to calculate the number density of water molecules throughout the channel structure. It is from this value that the related water free energy profile can then be estimated using Boltzmann inversion.

When the input to CHAP is an MD trajectory, the above procedure is repeated independently for each frame. CHAP will then calculate summary statistics (mean, standard deviation, minimum, and maximum) over time for each point in the channel profiles. The following sections now describe these different analysis steps in more detail.

## Pore Geometry and Center Line

The path-finding algorithm used by CHAP is based on a hard sphere representation of the protein, in which each atom is associated with a sphere of radius *r*_*i*_ centered at the position of the atom, **Q**_*i*_. Figure 5A illustrates how a pathway is found by squeezing a spherical probe through this collection of van der Waals spheres. Starting from an initial position inside the channel pore, **P**_0_, the probe is moved in steps of Δ*v* along the direction of a unit vector **e**_*v*_ pointing in the overall direction of the permeation pathway:

(1) $${\tilde{P}}_{i}=P_{i-1}+\Delta ve_{v}$$

The resulting point is then used to initialise a numerical optimization procedure seeking to maximize the radius of the probe,

(2) $$R\left(P\right)=\min_{j\in \left[1,N\right]}\|Q_{j}-P\|-r_{j}$$

without overlapping the van der Waals sphere of any protein atom. This optimization procedure is carried out in the plane supported by ${\tilde{P}}_{i}$ and spanned by the unit vectors **e**_*u*_ and **e**_*w*_ which are chosen to be perpendicular to both **e**_*v*_ and one another. The optimal probe position

(3) $$P_{i}=\arg\;\max_{u,w}R\left(\tilde{P}+ue_{u}+we_{w}\right)$$

is accepted as a point on the pore center line and a further step in the direction of **e**_*v*_ is carried out. This procedure is repeated until the probe radius exceeds a threshold, at which point it is assumed that the probe has left the pore. Subsequently, the iteration is carried out a second time moving in the direction of −** e**_*v*_ and starting from point **P**_1_.

In the original algorithm used by HOLE, the optimization of the probe position was performed by means of simulated annealing [5]. However, being a global optimization procedure, simulated annealing is prone to allowing the probe to jump out of the protein pore. The downhill simplex algorithm [34] was therefore implemented in CHAP alongside the simulated annealing procedure and this latter method was found to be generally more robust.

This procedure has three free parameters: The probe step size, Δ*v*, the number of Nelder–Mead iterations, *N*_simplex_, and the initial probe position, *P*_0_. For a sufficiently symmetric protein with a central pore, *P*_0_ can simply be set to the center of geometry of the overall channel protein. The step size needs to be small enough to resolve all structural features of interest. As illustrated in Fig. 5F, a probe step of 0.1 nm (the van der Waals radius of a hydrogen atom) is sufficient and the radius profile does not change significantly when the probe step is decreased further. Figure 5E shows the dependence of the error in the radius profile on the number of simplex iterations and reveals that 100 steps are sufficient to converge the profile to within a tolerance of ∼ 0.001 nm even for a very small probe step. This is the case not just for this particular 5-HT_3_ receptor structure, but also for a wide range of other ion channel proteins (Fig. S2).

The points ***P***_*i*_ on the center line are then connected into a continuous spatial curve ***S***(*s*) by means of B-spline interpolation as detailed in the Supplemental Information so that the curve passes exactly through all center line points:

(4) $$S\left(s_{i}\right)=P_{i}$$

A corresponding continuous radius profile is then found by interpolating the probe radii along the center line curve. The main advantage of a continuous center line curve over individual points is that the curve can be used to define a simple one-dimensional reaction coordinate for the motion of particles through the channel pore, as is detailed in the following section. In addition, it can be used to generate a pore surface representation through the path extrusion technique as shown in Fig. 1D. Importantly, for radially symmetrical pores, the radius profiles calculated using CHAP are almost identical to those calculated using HOLE (Fig. S3). However, it should be noted that the *s*-coordinate calculated by CHAP follows the pathway of the pore, and so for non-linear pathways this may differ from the *z*-coordinate calculated by HOLE.

## Water Density and Free Energy

We have previously shown that water density can be used to estimate a free energy profile for pore hydration and how this acts as proxy for measurement of ion permeation [22]. To achieve this, the center line spline is used to define a curvilinear coordinate system, in which the position of each particle is described by its distance from the spline curve, *ρ*, the arc length parameter value corresponding to the closest point on the spline curve, *s*, and the angle, *ϕ*, enclosed between the vector connecting this point and the particle position and the normal vector of the spline curve (Fig. 6A). The resulting coordinate system can be viewed as a polar version of the Frenet-Serret frame, providing a generalization of the canonical cylindrical coordinate system, where the *z*-coordinate is replaced by the length along the arc of the center line.

Unfortunately, there is no analytical expression relating the Cartesian coordinates of a particle to the curvilinear center line coordinates and this mapping has to be accomplished numerically by finding the minimal distance between the position of a particle and the spline curve. Given the position of the *j*-th particle in Cartesian coordinates, ***Q***_*j*_ = (*x*_*j*_, *y*_*j*_, *z*_*j*_)^⊤^, the corresponding spline coordinates are found from

(5) $$\rho_{j}=\min_{s}\|S\left(s\right)-Q_{j}\|$$

(6) $$s_{j}=\arg\;\min_{s}\|S\left(s\right)-Q_{j}\|-\arg\;\min_{s}\|S\left(s\right)-P_{0}\|$$

(7) $$\phi_{j}=\frac{\left(Q_{j}-S\left(s_{j}\right)\right)\cdot N}{\left\|Q_{j}-S\left(s_{j}\right)\|\;\|N\right\|}$$

where the minimization problem is solved using the method of Ref. [35]. Note that in the definition of the *s*-coordinate the projection of the initial probe position is subtracted. This ensures that the offset of the *s*-coordinate is consistent across multiple trajectory frames by setting it to zero for the initial probe position (which typically corresponds to the center of geometry of the protein).

The *s*-coordinate can be used as a one-dimensional collective variable along which to determine water density and the corresponding free energy profile. The probability density *P*(*s*, *ρ*, *ϕ*) of finding a water molecule at the given center line coordinates is given by the Boltzmann relation,

(8) $$P\left(s,\rho ,\phi \right)=\frac{1}{Z}\exp\;\left(-\mathrm{\beta G}\left(s,\rho ,\phi \right)\right)$$

where *G*(*s*, *ρ*, *ϕ*) is the Gibbs free energy, *Z* is the partition function, and *β*^−1^ = *k*_B_*T* with *k*_B_ being the Boltzmann constant and *T* the simulation temperature. In order to obtain a one-dimensional free energy profile, that is, *G*(*s*, *ρ*, *ϕ*) = *G*(*s*), the lateral degrees of freedom are integrated out according to

(9) $$P\left(s\right)=\int_{0}^{2\pi }\int_{0}^{R\left(s\right)}P\left(s,\rho ,\phi \right)\mathrm{\rho d\rho d\phi}=\frac{2\mathrm{\pi R}{\left(s\right)}^{2}}{Z}\exp\;\left(-\mathrm{\beta G}\left(s\right)\right)$$

where it is assumed that the cross-sectional area of the pore is approximately circular with a local radius *R*(*s*). Inverting the above relationship yields an expression for the free energy of the water molecule,

(10) $$G\left(s\right)=-\frac{1}{\beta }\left(\ln\;\frac{P\left(s\right)}{\mathrm{\pi R}{\left(s\right)}^{2}}+\ln\;Z\right)$$

into which the partition function enters only as an additive constant. This constant is determined by the convention that the free energy in the bulk outside the protein should be zero, that is, *G*(*s*) → 0 as *s* →  ± ∞.

The probability density appearing in the above expression can be estimated directly from the MD trajectory by means of kernel density estimation:

(11) $$P_{t}\left(s\right)=\frac{1}{N}\sum_{i=1}^{N}K_{h}\left(s,s_{i}\left(t\right)\right)$$

Here *s*_*i*_(*t*) is the position of the *i*-th water molecule at time *t*, *N* is the overall number of water molecules, and *P*_*t*_(*s*) denotes the probability density estimated at time *t*. A Gaussian kernel of the form

(12) $$K_{h}\left(s,s_{i}\right)=\frac{1}{\sqrt{2\pi }h}\exp\;\left(-\frac{s-s_{i}}{2h^{2}}\right)$$

is employed here. A conceptual representation of kernel density estimation is shown in Fig. 6B.

The bandwidth *h* is a free parameter in kernel density estimation that fulfills a function similar to the bin width in histograms and care must be taken when selecting a value for this parameter as the resulting density profile is sensitive to it. A value for this parameter of 0.24 nm has been suggested for identifying liquid interfaces in three dimensions [36], whereas a value of 0.1 nm has been used in a study of pore de-wetting in the bacterial GLIC channel [17]. It is also possible to estimate an appropriate bandwidth directly from the data [33]. This method minimizes the asymptotic mean integrated square error (AMISE) of the density estimate.

**Fig. 7:**
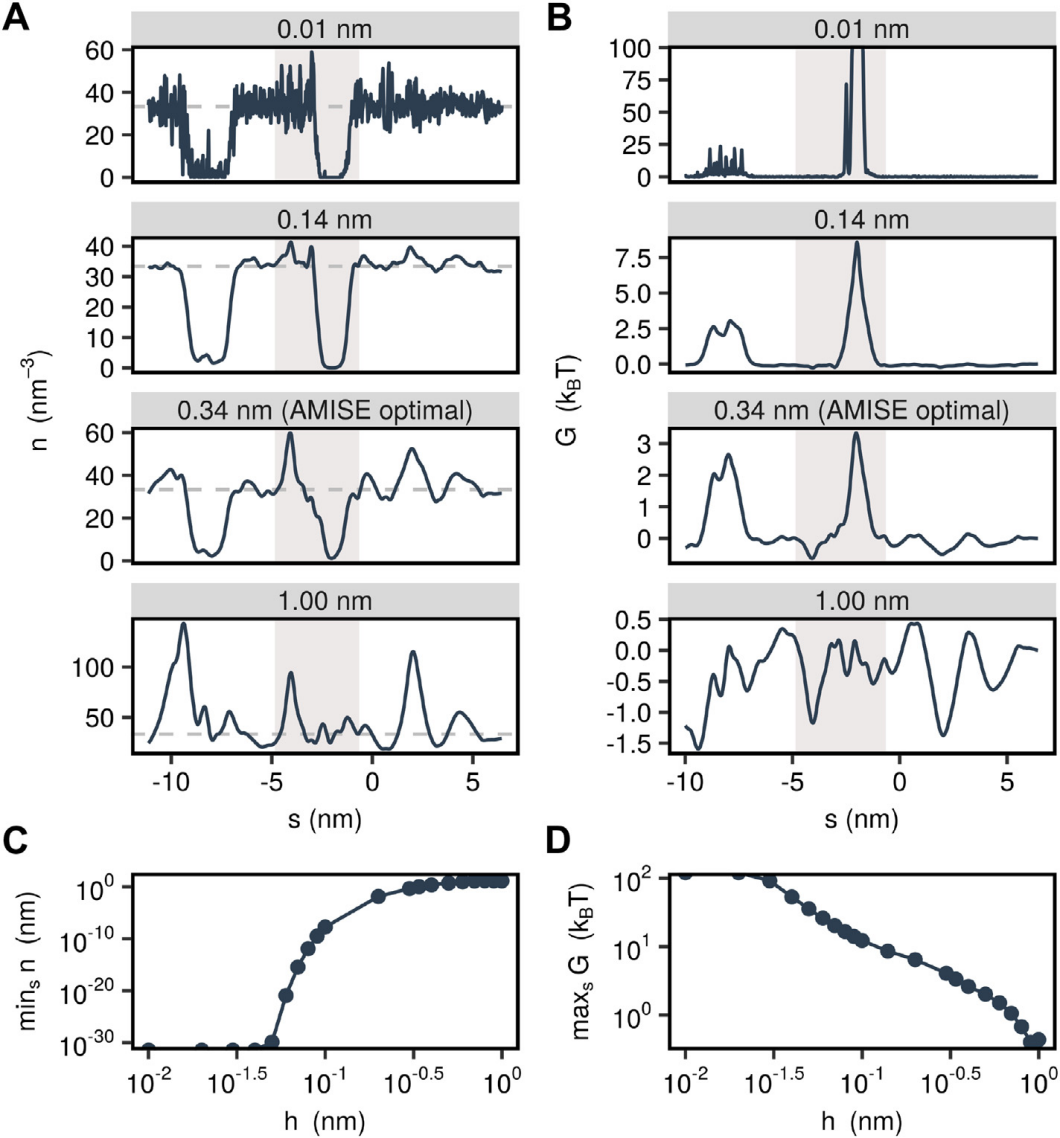
Influence of bandwidth parameter. (A) Water number density along the permeation pathway of the 5-HT_3_ receptor for various values of the kernel bandwidth. A value of 0.14 nm corresponds to the approximate size of a water molecule and a value of 0.34 nm corresponds to the statistically optimal bandwidth. (B) Corresponding free energy profiles calculated via Boltzmann inversion of the density profiles. The gray shaded area in panels A and B represents the transmembrane domain. (C) Minimum of number density profile in dependence of bandwidth. (D) Maximum of free energy profile in dependence of bandwidth.

Figure 7 uses the example of the 5-HT_3_ receptor to illustrate how the water density and free energy profiles depend on the bandwidth parameter. For very small values of *h*, the water density estimate is very noisy and in low density regions it drops down to zero, so that the corresponding free energy at these points is singular. Conversely, if a very large bandwidth is chosen, the estimate of *P*(*s*) is near uniform throughout the pore and the water density estimate exhibits over-densities at points where the pore radius is particularly small. In this case, the free energy profile exhibits no clear barriers or wells.

The AMISE-optimal bandwidth, which in this case is 0.34 nm, finds a balance between these two extremes. The overall density profile is free of noise and the lowered water density in the region of the hydrophobic gate is readily apparent. However, there are still some regions where the water density is unphysically large. By comparison, a profile estimated using a bandwidth of 0.14 nm (the approximate radius of a water molecule) yields a water density that never much exceeds the experimental bulk density of water while still showing little sign of noise. Using a bandwidth fixed at the size of a water molecule also permits a more direct comparison between different channel structures, while the AMISE-optimal bandwidth may differ across of different overall pore volume. It is therefore recommended to use a default value of 0.14 nm.

## Hydrophobicity Profiles

In addition to calculating the channel radius and water density profiles inside the pore, it is also necessary to quantify the hydrophobicity along the conduction pathway. For cylindrical nanopores, it has been found that the free energy difference between hydrated and dehydrated pore states is given by

(13) $$\Delta \Omega =\Omega_{\text{vapor}}-\Omega_{\text{liquid}}=2\mathrm{\pi R}\gamma_{\mathrm{lv}}\left(R+L\cos\;\left(\theta_{e}\right)\right)$$

where *R* and *L* are the radius and length of the nanopore, *γ*_lv_ is the liquid–vapor surface tension of water, and *θ*_*e*_ is the contact angle from the Young–Dupré Equation [13]. Determining a hydrophobicity profile for a channel therefore requires knowledge about the amino acid residues facing a given section of the pore and their associated contact angle. The individual hydrophobicity values at the positions of the amino acids can then be combined into a continuous hydrophobicity profile by means of kernel smoothing.

CHAP can automatically identify pore-facing residues, that is, residues exposing their amino acid side chains to the channel pore, via a two-step procedure. First, all residues whose center of geometry lies within a given cutoff, *R*_thres_, from the pore surface are determined. To accomplish this, the position of the center of geometry of each residue is mapped from Cartesian coordinates to center line spline coordinates so that each residue is associated with a distance from the center line, *ρ*_*i*_^cog^, and a distance along the center line, *s*_*i*_^cog^, where the index runs over all protein residues. If

(14) $$\rho_{i}^{\mathrm{cog}}\gg R(s_{i}^{\mathrm{cog})}+R_{\text{thres}}$$

the *i*-th residue will be considered pore-lining, where *R*(*s*_*i*_^cog)^ is the pore radius at the position of the residue. A typical threshold value for an *α*-helical pore is ∼ 0.75 nm.

**Fig. 8:**
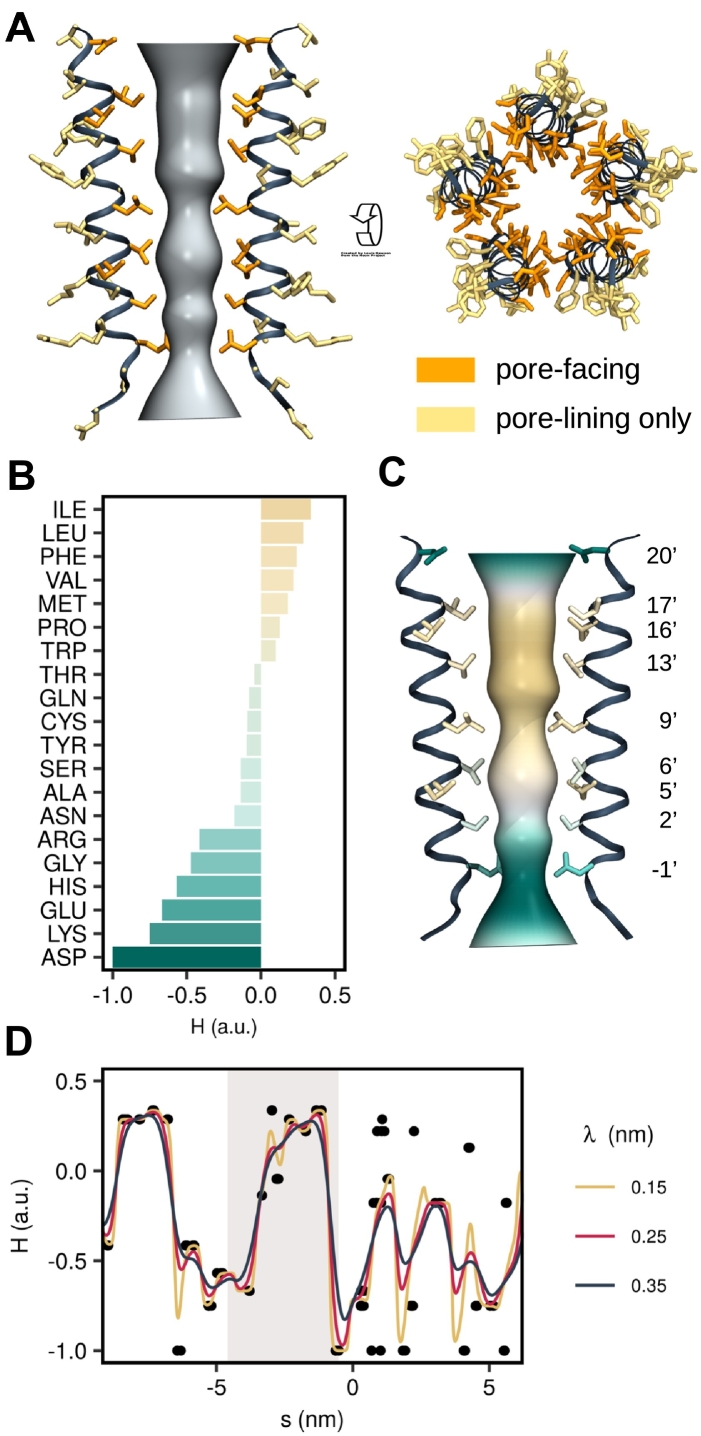
Hydrophobicity profile of pore-lining residues. (A) CHAP automatically subdivides the pore-lining residues into two categories: those whose side chains directly face the pore to influence the permeation pathway (i.e., pore-facing residues), and those whose side chains are close to, but point away from the pore (i.e., pore-lining only). In this example of the M2 helix bundle of the 5-HT_3_ receptor TMD, the “pore-facing” residues are shown in orange, while the “pore-lining only” residues are shown in yellow. (B) Amino acid hydrophobicities according to the scale proposed by Wimley and White. In CHAP, the original hydrophobicities are rescaled into the interval from − 1 (very hydrophilic) to + 1 (very hydrophobic) to facilitate an easy comparison between different hydrophobicity scales. (C) Permeation pathway colored according to the Wimley–White hydrophobicity of the pore-facing residues. (D) Hydrophobicity profile of the entire 5-HT_3_ receptor pore. Black circles represent the position and hydrophobicity of the pore-facing residues (averaged over all five subunits). Lines represent the kernel-smoothed continuous hydrophobicity profile underlying the surface coloring in panel C. Different values of the smoothing span influence the exact magnitude of hydrophobicity fluctuations, but the overall distinction between hydrophobic and hydrophilic regions along of the pore remains unaffected. The shaded area corresponds to the transmembrane region shown in panels A and C.

Second, for each pore-lining residue, the position of the *α*-carbon is also mapped to pathway coordinates *ρ*_*i*_^*α*^ and *s*_*i*_^*α*^. If the distance from the *α*-carbon to the pore center line is larger than the distance between the center of geometry of the overall residue and the pore center line, that is, if

(15) $$\rho_{i}^{\alpha }\gg \rho_{i}^{\mathrm{cog}}$$

then the *i*-th residue will be considered pore-facing. The property of being pore-lining and pore-facing is encoded in two binary indicator variables, whose values are written to the temperature factor and occupancy fields of a PDB file of the input structure to allow easy visualization of these residues. If CHAP is run with a trajectory as input, the above analysis is performed repeatedly (once for each frame) and the values are written to the PDB file correspond to the time average of the indicator variables. Figure 8A illustrates the distinction between pore-lining and pore-facing residues in the 5-HT_3_ receptor.

To the best of our knowledge, no experimental data quantifying the contact angle at the interface between water and specific amino acids are available. However, various amino acid hydrophobicity scales have been proposed in the literature. An early hydropathy scale based on a collection of experimental observations has been suggested by Kyte *et al*. [37], while another has been based on measurements of the stability of *α*-helices [38]. Other scales have used the free energy of partitioning of oligopeptides between water and a lipid environment [39], [40], while some report free energies of transferring amino acids from water into a lipid bilayer [41]. A biological scale has also been proposed based on the recognition of *α*-helices by the endoplasmic reticulum translocon [42], while another is derived from MD simulations of a water nanodroplet on a planar layer of amino acids [43].

For comparability, the hydrophobicity scales listed above have been shifted and scaled to the interval ranging from − 1 for the most hydrophilic to + 1 for the most hydrophobic residues while preserving their natural zero (Fig. S4). Note that this contrasts with the physical definition of hydrophobicity in terms of cos *θ*_*e*_ and instead follows the biochemical convention of associating a larger value with a more hydrophobic residue. While the exact hydrophobicity value assigned to each amino acid differs to some degree across scales (Fig. S4), we found no marked difference in their ability to predict pore de-wetting in a recent analysis of nearly 200 ion channel structures [44]. By default, CHAP employs the Wimley and White scale [40], but users are free to select an alternative scales that may be more appropriate for their structure.

CHAP uses the normalized hydrophobicity scales to associate a hydrophobicity value, *H*_*i*_, with each residue position *s*_*i*_^cog^. A continuous hydrophobicity profile is then calculated from these discrete hydrophobicity values using a Nadaraya–Watson kernel-weighted average. Mathematically this is expressed as

(16) $$H\left(s\right)=\frac{\sum_{i}K_{\lambda }\left(s,s_{i}\right)H_{i}}{\sum_{i}K_{\lambda }\left(s,s_{i}\right)}$$

where

(17) $$K_{\lambda }\left(s,s_{i}\right)=\exp\;\left(-\frac{{\left|s-s_{i}\right|}^{2}}{2\lambda^{2}}\right)$$

is a Gaussian kernel. The smoothing span *λ* is chosen to reflect the finite extension of an amino acid residue. Since the pitch of an *α*-helix is approximately 0.15 nm per residue and on average every third residue is pore-facing, a value of ∼ 0.225 nm (for a width of the Gaussian kernel of ∼ 0.45 nm) can be considered reasonable. Note that far away from any residues (e.g., in the bulk water region outside the channel pore) *H*(*s*) will decay exponentially to zero.

In Fig. 8D, this is illustrated on the 5-HT_3_ receptor using the Wimley and White hydrophobicity scale. As can be seen, there is a considerable increase in hydrophobicity in the region of the transmembrane domain containing the hydrophobic gate. This section of the profile is shown in Fig. 8C mapped onto a 3D representation of the pore together with the M2 helices of the 5-HT_3_ receptor, illustrating how the hydrophobicity of the pore-facing residues influences the hydrophobicity profile.

## Conclusion

In this study, we present a versatile computational tool for the functional annotation of ion channel structures: the Channel Annotation Package, CHAP. This tool implements a methodology for predicting the conductive state of ion channel structures that goes beyond a measurement of the pore radius to include profiles of pore hydrophobicity as well as of water density and free energy based on MD simulations of water behavior within an experimentally determined channel structure. Crucially, this enables CHAP to identify hydrophobic gates that cannot be detected using methods based purely on measuring the physical dimensions of the pore alone.

Importantly, the analysis performed by CHAP is computationally inexpensive compared to more complex simulation techniques such as computational electrophysiology [45] and is therefore highly suitable for the high-throughput analysis of the rapidly growing number of ion channel structures. The utility of this approach is demonstrated in a recent study [44] employing CHAP to investigate the prevalence of hydrophobic gates in 200 different ion channel structures.

CHAP is made available as free and open-source software. Copies of the source code can be obtained from www.channotation.org and include scripts for visualizing results in the molecular graphics programs VMD [46] and PyMOL [47].

## Appendix A Supplementary data

Supplementary materials Image 1
