# Supplementary material for: CHAP: A Versatile Tool for the Structural and Functional Annotation of Ion Channel Pores
Source: J Mol Biol. 2019 Aug 9;431(17):3353–65. doi: 10.1016/j.jmb.2019.06.003 (PMC6699600; doi:10.1016/j.jmb.2019.06.003)
Supplement: Supplementary file 1 — Supplementary materials [file mmc1.pdf]

Supplemental information for CHAP: A versatile tool for the structural and functional annotation of ion channel pores

Molecular Dynamics Simulation Protocol

Equilibrium molecular dynamics simulations of the 5-HT_3 receptor were performed using GROMACS 2018. Starting structures were generated from the original crystal structure (PDB ID: 4PIR) with missing atoms being added by the WHAT IF tool [1]. This starting structure was embedded in a DOPC bilayer using an established serial multiscale protocol [2]. The resulting protein-bilayer system was converted back to an atomistic representation and solvated in $150\,\text{mmol}\,\text{L}^{-1}$ NaCl solution. The CHARMM36 all-atom force field [3] was used together with the TIP3P model of water [4]. Simulations of the TRPV4 channel (PDB ID: 6BBJ) followed the same protocol.

Long-range electrostatic interactions are treated using the particle mesh Ewald (PME) method [1, 5] employing a short range cutoff of 1 nm and a Fourier spacing of 0.12 nm. To permit subsequent determination of the Gibbs free energy from the water molecule distribution, the system is simulated in the isothermal-isobaric (NPT) ensemble at a temperature of 310 K and a pressure of 1 bar. A v-rescale thermostat [6] with a coupling constant of 0.1 ps is used for temperature control and a pressure is maintained semi-isotropically using the method of [7] with a coupling constant of 1 ps.

The canonical leapfrog method with a time step of 2 fs is used to integrate three independent copies of the system for 100 ns with bonds constrained through the linear constraints solver algorithm LINCS [8]. Additionally, the Cα atoms of all protein residues are placed under a harmonic restraint with a force constant of $1000\,\text{kJ/mol/nm}^2$ to ensure that the configuration of the simulated channel does not deviate from the experimentally determined structure. A simulation length of 100 ns likely exceeds that required, since the largest permitted protein motions are side chain reorientations, which typically occur on a time scale of ~ 10 ns and most pore dewetting events appear to occur even more rapidly. For the examples described in this study, analysis of three 30 ns long simulations would produce similar results.

B-Spline Curves

Formally, a spline curve of degree p along some parameter s can be written as the linear combination

$$\tilde{\mathbf{S}}_p(\tilde{s}) = \sum_{k=1}^{N} \mathbf{c}_k B_{k,p}(\tilde{s}) \tag{1}$$

where

$$B_{k,p}(\tilde{s}) = \frac{\tilde{s} - t_k}{t_{k+p} - t_k} B_{k,p-1}(\tilde{s}) + \frac{t_{k+p+1} - \tilde{s}}{t_{k+p+1} - t_{k+1}} B_{k+1,p-1}(\tilde{s}) \tag{2}$$

with $B_{k,0}(\tilde{s}) = 1$ if $t_k \leq \tilde{s} \leq t_{k+1}$ and $B_{k,0}(\tilde{s}) = 0$ otherwise are the recursively defined B-spline basis functions. Here $\{\mathbf{c}_k\}_{k=1}^{N}$ denotes the spline curve control points and $\{t_k\}_{k=1}^{N+p+1}$ is referred to as knot vector. Note that a zero division in the above equations is treated as $0/0 = 0$.

In order to find the spline curve that interpolates the centre line points $\mathbf{P}_i$ from the pore finding algorithm, one demands

$$\mathbf{S}_p(\tilde{s}_i) = \mathbf{P}_i \tag{3}$$

which requires a parameterisation of the discrete point set. Ideally, this should be the distance between the points along the arc of the spline curve, but unfortunately this distance is not known a priori. Instead, the commonly applied chord length approximation is used here, in which points are parameterised by their Euclidean distance according to:

$$\tilde{s}_{i+1} = \tilde{s}_i + ||\mathbf{P}_{i+1} - \mathbf{P}_i|| \tag{4}$$

Importantly, it has been also shown that this choice of parameterisation guarantees full approximation order for spline curves up to third degree [9].

For a cubic spline curve $\mathbf{S}(\tilde{s})$, where the subscript $p = 3$ has been dropped for clarity, Equation 3 together with the knot vector

$$\{t_k\}_{k=1}^{N+p+1} = \{\tilde{s}_1, \tilde{s}_1, \tilde{s}_1, \tilde{s}_1, \tilde{s}_2, \tilde{s}_3, \ldots, \tilde{s}_{N-1}, \tilde{s}_N, \tilde{s}_N, \tilde{s}_N, \tilde{s}_N\} \tag{5}$$

and hermite boundary conditions then leads to a tridiagonal linear system, from which the control points can be determined efficiently through the Thomas algorithm. The choice of cubic splines is motivated by the fact that cubic splines are twice continuously differentiable so that the curvature of the resulting spline curve is guaranteed to be continuous. At the same time, cubic splines are known to minimise overall curvature, preventing undue variation of the interpolant between the given centre line points.

The resulting spline curve is subsequently re-parameterised by arc length using a three-step procedure proposed by [10]. In a first step, the arc length in each interval is computed according to

$$l_k = \int_{\tilde{s}_k}^{\tilde{s}_{k+1}} ||\tilde{\mathbf{S}}(\tilde{s})|| d\tilde{s} = \int_{\tilde{s}_k}^{\tilde{s}_{k+1}} \sqrt{\left(\tilde{\mathbf{S}}'(\tilde{s})\Big|_x\right)^2 + \left(\tilde{\mathbf{S}}'(\tilde{s})\Big|_y\right)^2 + \left(\tilde{\mathbf{S}}'(\tilde{s})\Big|_z\right)^2} d\tilde{s} \tag{6}$$

from which the overall length of the spline curve between the two openings of the pore can be calculated as:

$$L = \sum_{k=1}^{N-1} l_k \tag{7}$$

The integral in the expression for l_k is solved numerically through sixth-order accurate Newton-Cotes quadrature (Boole's rule).

The second step comprises calculating the chord length parameter values $\tilde{s}_m$ corresponding to the equidistant arc length parameter values $s_m = m\Delta s = mL/M$ with $m \in [0, M]$. This is equivalent to finding the root of

$$m\Delta s - \left(\sum_{k=1}^{q} l_k + \int_{\tilde{s}_q}^{\tilde{s}_m} ||\tilde{\mathbf{S}}'(\tilde{s})|| \; d\tilde{s} \right) = 0 \tag{8}$$

where $q \in [1, N]$ is the index of the spline interval for which

$$\sum_{k=0}^{q} l_k \leq m\Delta s < \sum_{k=0}^{q+1} l_k. \tag{9}$$

The root-finding problem is solved through the algorithm of [11], which is known to be the asymptotically most efficient root-finding method.

In the final step, the interpolation problem

$$\mathbf{S}(s_m) = \tilde{\mathbf{S}}(\tilde{s}_m) \tag{10}$$

is solved to obtain a new spline curve, $\mathbf{S}(\mathbf{s})$, that approximates the original curve, but is parametrized by arc length rather than chord length. This curve is then used to describe the centre line of the channel pore.

References

[1] G. Vriend, WHAT IF: A molecular modeling and drug design program, J. Mol. Graph. 8 (1) (1990) 52–56.

[2] P. J. Stansfeld, M. S. P. Sansom, From coarse grained to atomistic: a serial multiscale approach to membrane protein simulations, J. Chem. Theory Comput. 7 (4) (2011) 1157–1166.

[3] J. Huang, A. D. MacKerell, Jr, CHARMM36 all-atom additive protein force field: validation based on comparison to NMR data, J. Comput. Chem. 34 (25) (2013) 2135–2145.

[4] W. L. Jorgensen, J. Chandrasekhar, J. D. Madura, R. W. Impey, M. L. Klein, Comparison of simple potential functions for simulating liquid water, J. Chem. Phys. 79 (2) (1983) 926–935.

[5] T. Darden, D. York, L. Pedersen, Particle mesh ewald: An N·log(N) method for ewald sums in large systems, J. Chem. Phys. 98 (12) (1993) 10089–10092.

[6] G. Bussi, D. Donadio, M. Parrinello, Canonical sampling through velocity rescaling, J. Chem. Phys. 126 (1) (2007) 014101.

[7] M. Parrinello, A. Rahman, Polymorphic transitions in single crystals: A new molecular dynamics method, J. Appl. Phys. 52 (12) (1981) 7182–7190.

[8] B. Hess, H. Bekker, H. J. C. Berendsen, Johannes G E, LINCS: A linear constraint solver for molecular simulations, J. Comput. Chem. 18 (12) (1997) 1463–1472.

[9] M. S. Floater, T. Surazhsky, Parameterization for curve interpolation, in: Studies in Computational Mathematics, 2006, pp. 39–54.

[10] H. Wang, J. Kearney, K. Atkinson, Arc-length parameterized spline curves for real-time simulation, in: Proc. 5th International Conference on Curves and Surfaces, 2002, pp. 387–396.

[11] G. E. Alefeld, F. A. Potra, Y. Shi, Algorithm 748; enclosing zeros of continuous functions, ACM Trans. Math. Softw. 21 (3) (1995) 327–344.

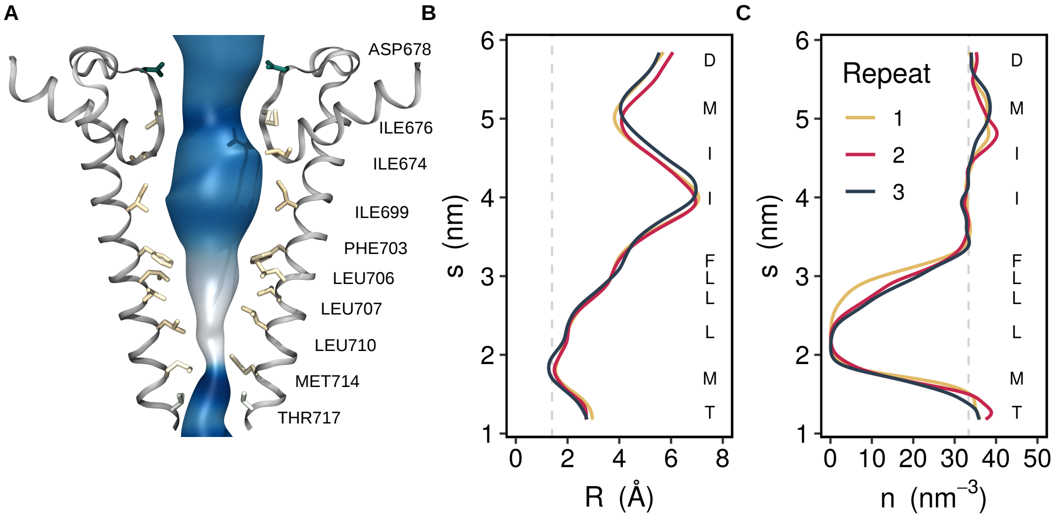

Figure S1: A hydrophobic gate in TRPV4. (A) Permeation pathway through the transmembrane domain of the TRPV4 channel. The pathway was calculated on a snapshot from a simulation based on the PDB structure 6BBJ. Pore-facing residues are shown in licorice representation and are coloured according to their hydrophobicity. (B) Time-averaged radius profile of the permeation pathway determined from three independent MD simulations. (C) Corresponding water number density profiles.

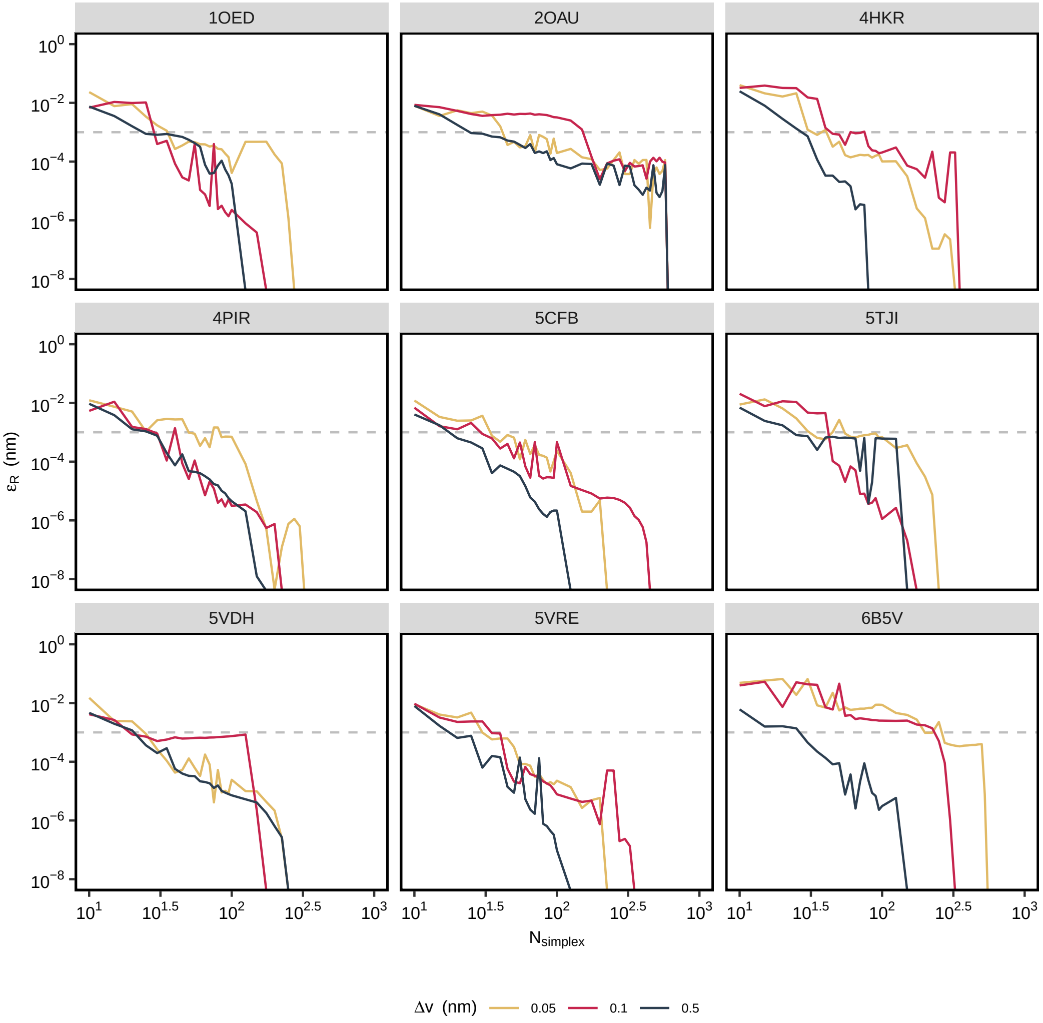

Figure S2: Convergence of the Nelder-Mead algorithm. The error of the radius profile calculated for nine different channel structures using three different values for the probe step is shown. Convergence to within 0.001 nm is typically reached within 100 iterations.

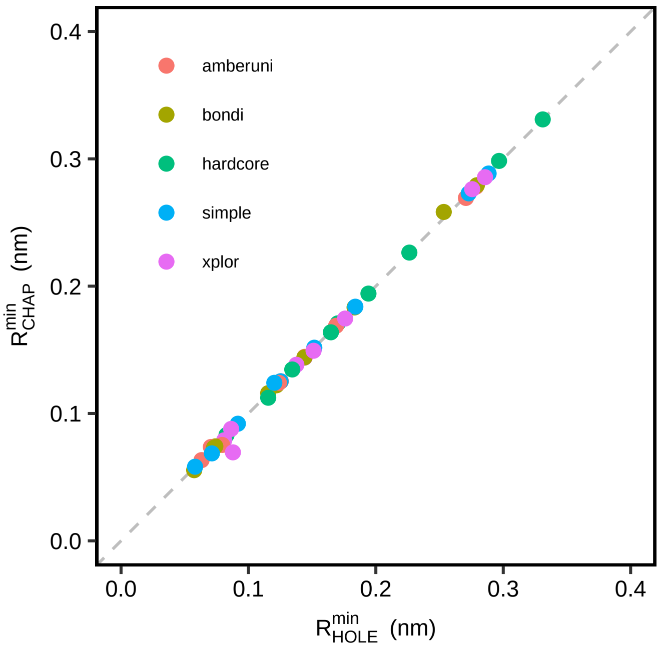

Figure S3: Comparison of the radius at the narrowest constriction between CHAP and HOLE. Calculated for nine representative ion channel structures using the five different van der Waals radius datasets available in HOLE. In all cases the radii agree to within 0.01 nm.

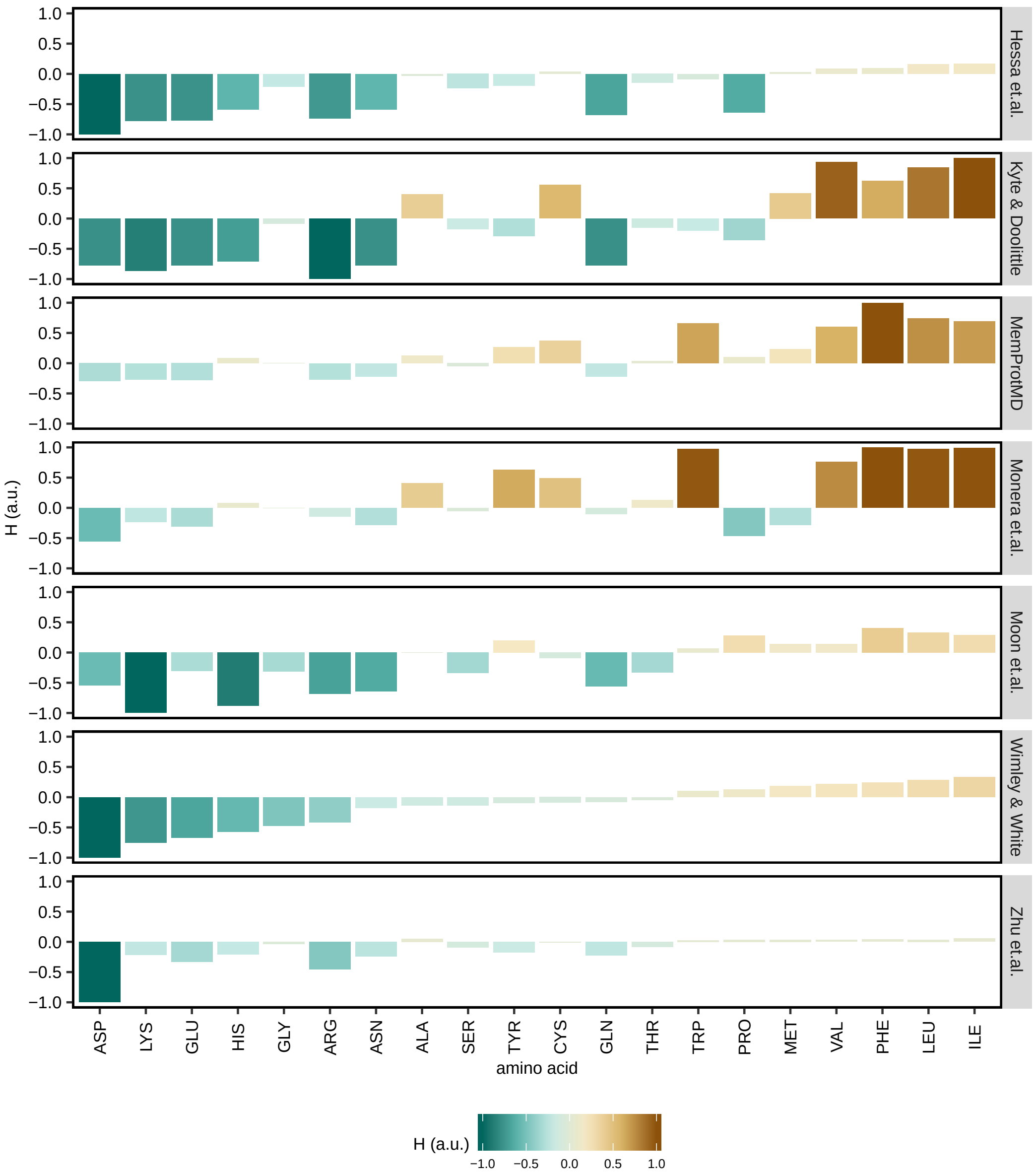

Figure S4: Comparison of amino acid hydrophobicity scales available in CHAP. The amino acids are listed in order of increasing hydrophobicity according to the Wimley-White scale, which is the default scale used by CHAP.
